# Supplementary material for: A high-resolution mRNA expression time course of embryonic development in zebrafish
Source: eLife. 2017 Nov 16;6:e30860. doi: 10.7554/eLife.30860 (PMC5690287; doi:10.7554/eLife.30860)
Supplement: Supplementary file 6. [file elife-30860-supp6.zip › biolayout-clusters-files/Cluster231-genes.html]

Cluster231


# Cluster231: Genes

| Ensembl ID | Gene Name | Chr | Start | End | Biotype |
| --- | --- | --- | --- | --- | --- |
| ENSDARG00000086764 | ENSDARG00000086764 | 16 | 10438799 | 10455227 | protein\_coding |
| ENSDARG00000102091 | ENSDARG00000102091 | 1 | 58621585 | 58629495 | protein\_coding |
| ENSDARG00000099640 | eed | 1 | 27690 | 34330 | protein\_coding |
| ENSDARG00000044299 | lmnb1 | 10 | 16078127 | 16098789 | protein\_coding |
| ENSDARG00000008551 | pole3 | 5 | 32287966 | 32290654 | protein\_coding |
| ENSDARG00000008936 | sec11a | 18 | 26805705 | 26815345 | protein\_coding |
